# Supplementary material for: Conserved human effector Treg cell transcriptomic and epigenetic signature in arthritic joint inflammation
Source: Nat Commun. 2021 May 11;12:2710. doi: 10.1038/s41467-021-22975-7 (PMC8113485; doi:10.1038/s41467-021-22975-7)
Supplement: Supplementary file 1 — Supplementary Information [file 41467_2021_22975_MOESM1_ESM.pdf]

Supplementary Information

Conserved human effector Treg cell transcriptomic and epigenetic signature in inflammation

Gerdien Mijnheer,[#] Lisanne Lutter,[#] Michal Mokry, Marlot van der Wal, Rianne Scholman, Veerle Fleskens, Aridaman Pandit, Weiyang Tao, Mark Wekking, Stephin Vervoort, Ceri Roberts, Alessandra Petrelli, Janneke G.C. Peeters, Marthe Knijff, Sytze de Roock, Sebastiaan Vastert, Leonie S. Taams, Jorg van Loosdregt,[†] Femke van Wijk[†*]

[#]These authors contributed equally

†These authors jointly supervised this work

*Corresponding author: f.vanwijk@umcutrecht.nl

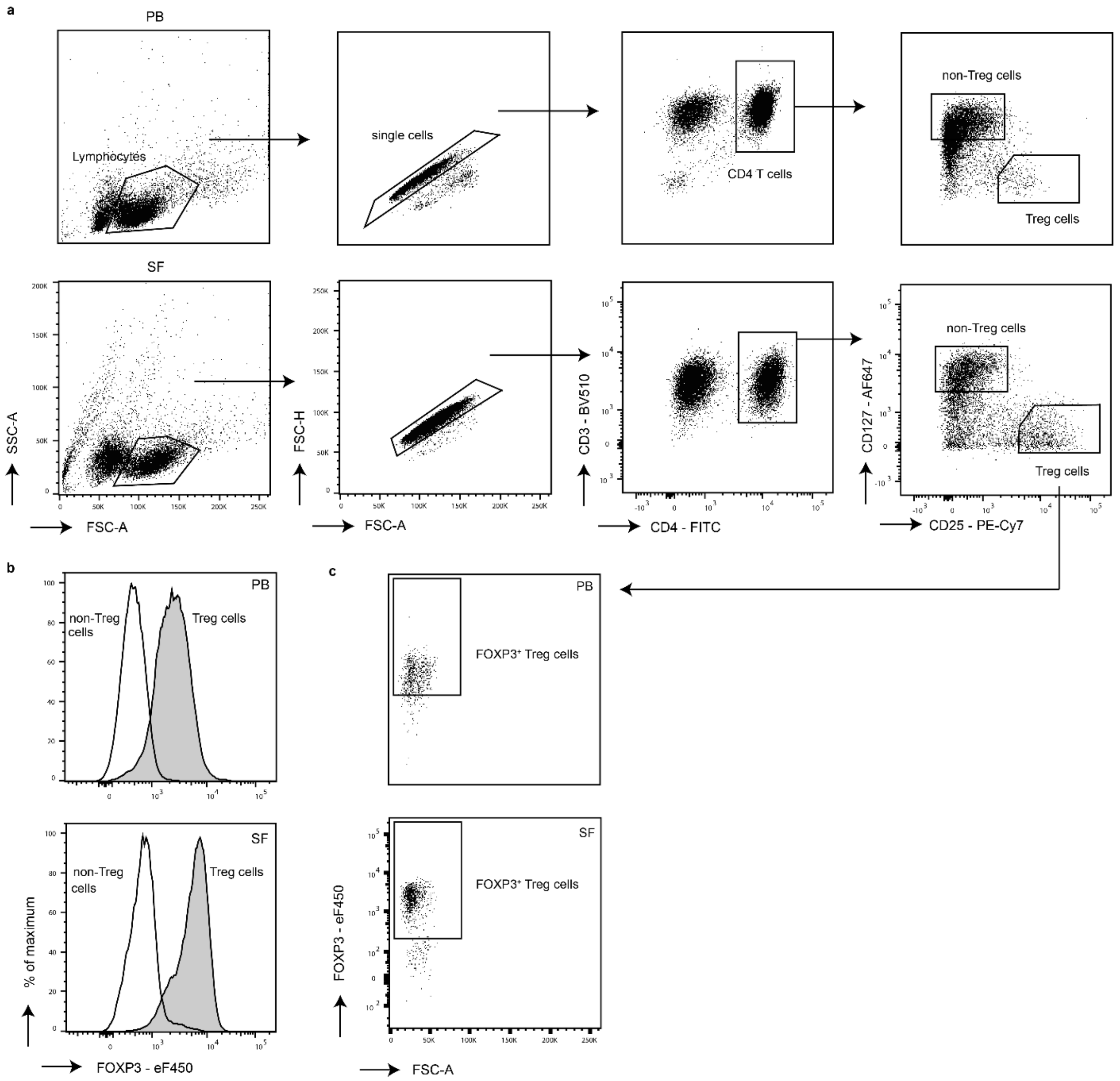

Supplementary Figure 1. Gating strategy and FOXP3 expression of sorted cells. a Representative gating strategy to sort and FACS both peripheral blood (PB)- and synovial fluid (SF)-derived Treg cells and non-Treg cells. **b** FOXP3 Median Fluorescence Intensity (MFI) plots of sorted SF- and PB-derived Treg cells and non-Treg cells. **c** Representative gating strategy of FOXP3 within Treg cells, gating as per **a**, in PB and SF. All panels are representative of two or more independent experiments.

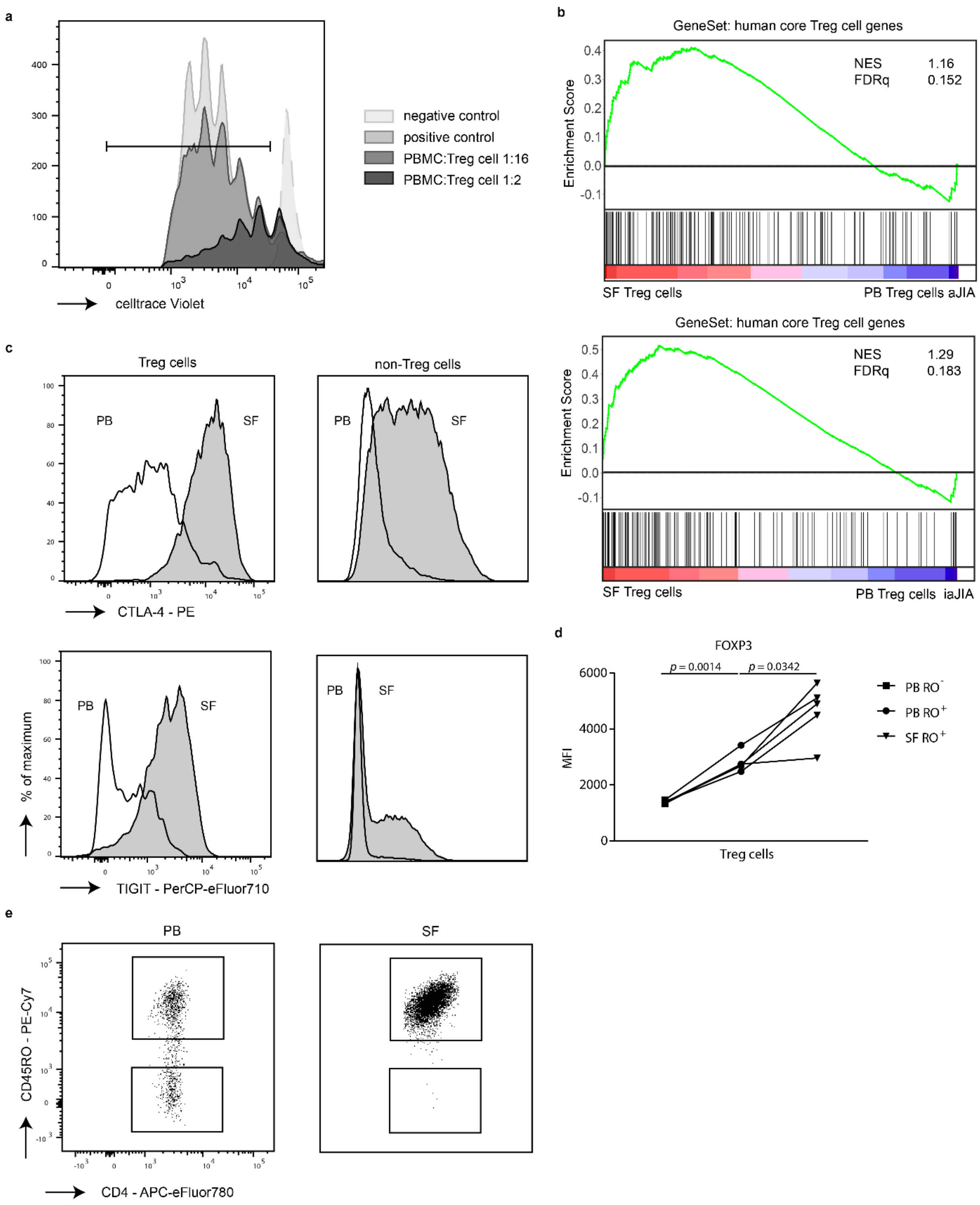

Supplementary Figure 2. GSEA and protein expression shows the Treg cell signature enriched in SF Treg cells. a Overview of the gating of proliferating peripheral blood mononuclear cells (PMBC), suppression assay as per Fig. 1b, without stimulation (negative control, dashed line), with stimulation (positive control, light gray fill), with 1:16 PBMC:Treg cells (dark gray fill) and with 1:2 PBMC:Treg cells (black fill). The bar shows the cells counted as proliferating. **b** GeneSet Enrichment Analysis (GSEA) of human core Treg cell signature genes (identified by Ferraro *et al.*[72]) in pairwise comparison of synovial fluid (SF) and peripheral blood (PB) Treg cells derived from juvenile idiopathic arthritis (JIA) patients with

active (left) or inactive (right) disease, represented by the normalized enrichment score (NES) and False Discovery Rate statistical value (FDRq, multiple hypothesis testing using sample permutation). **c** Representative MFI plots (for Fig. 1e) for both CTLA4 (top) and TIGIT (bottom) in Treg cells (left) and non-Treg cells (right) derived from paired SFMC (grey fill) and PBMC (no fill) of JIA patients. **d** Comparison of the MFI of FOXP3 within PB $CD45RO^-$ (PB RO^-, square, healthy adults), PB $CD45RO^+$ (PB RO^+, circle, patients with active JIA), and SF $CD45RO^+$ (SF RO^+, triangle, patients with active JIA) (n=5 per subset). Statistical comparisons were performed using one-way ANOVA with Tukey correction for multiple testing. **e** Representative gating strategy of $CD45RO^+$ and $CD45RO^-$ Treg cells in PB and SF for **d.** (**d** and **e**) Data are representative of two independent experiments. (**a** and **c**) The graphs are representative for two independent experiments with n=4 and n=5 total, respectively. Source data are provided as a Source Data file.

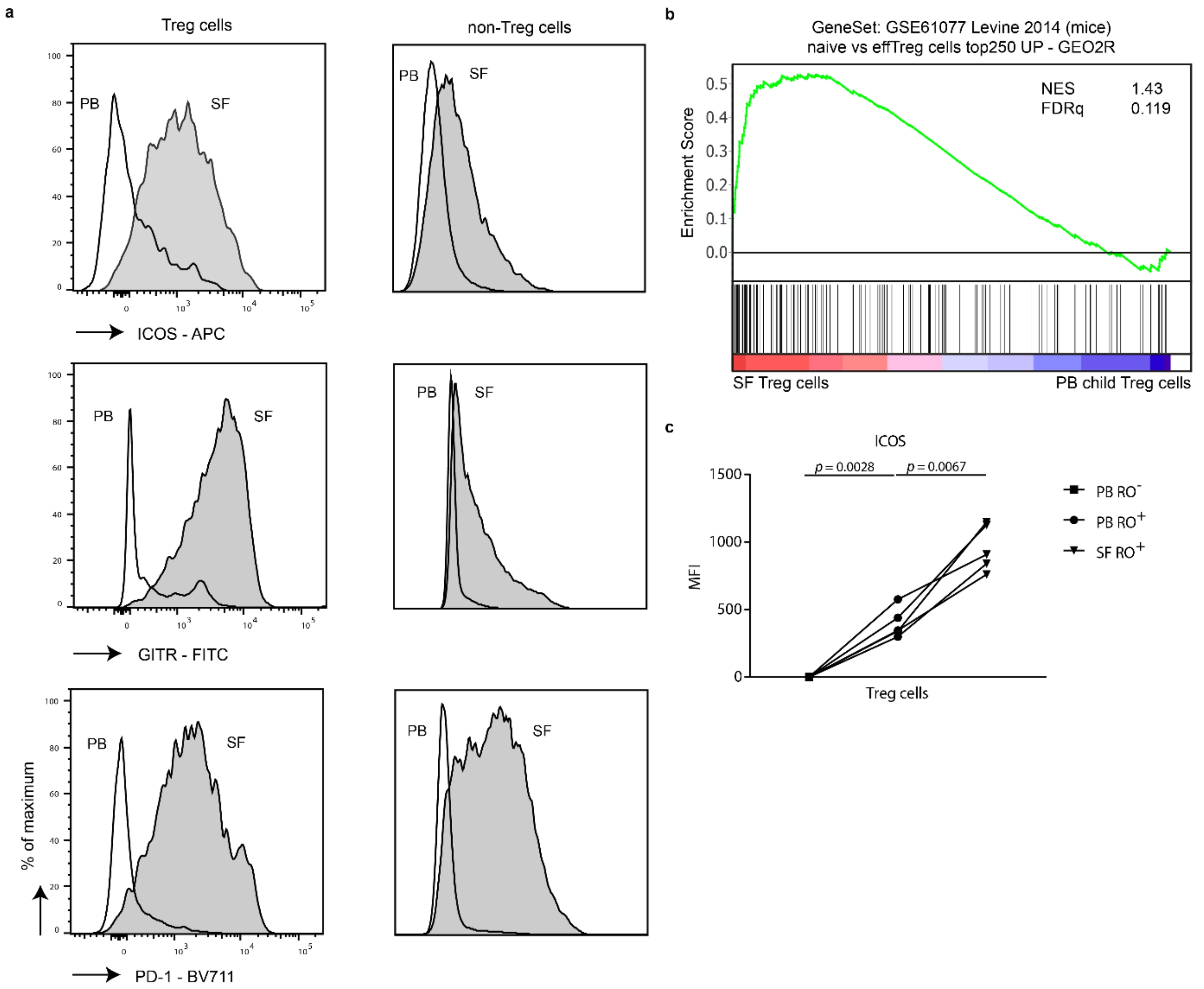

Supplementary Figure 3. GSEA and protein expression show an effector Treg cell signature in SF Treg cells. a Representative Median Fluorescence Intensity (MFI) plots (for Fig. 2c) of ICOS (top), GITR (middle) and PD-1 (bottom) in paired synovial fluid (SF)- and peripheral blood (PB)-derived Treg cells (top) and non-Treg cells (bottom) from juvenile idiopathic arthritis (JIA) patients. SF: grey fill, PB: no fill. The graphs are representative for two independent experiments with n=5 total. **b** GeneSet Enrichment Analysis (GSEA) of effector eTreg cell genes (as identified with GEO2R as the top250 genes upregulated in naïve versus eTreg cells, published in Levine *et al.*[21]) in pairwise comparison of SF (n=4) and healthy child PB (n=3) Treg cells, represented by the normalized enrichment score (NES) and FDR statistical value (FDRq, multiple hypothesis testing using sample permutation). **c** Comparison of the MFI of ICOS within PB CD45RO$^-$ (PB RO$^-$, square, healthy adults), PB CD45RO$^+$ (PB RO$^+$, circle, patients with active JIA), and SF CD45RO$^+$ (SF RO$^+$, triangle, patients with active JIA) (n=5 per subset). See Supplementary Figure 2d for the gating strategy of CD45RO$^{-/+}$ Treg cells. Statistical comparisons were performed using one-way ANOVA with Tukey correction for multiple testing. Source data are provided as a Source Data file.

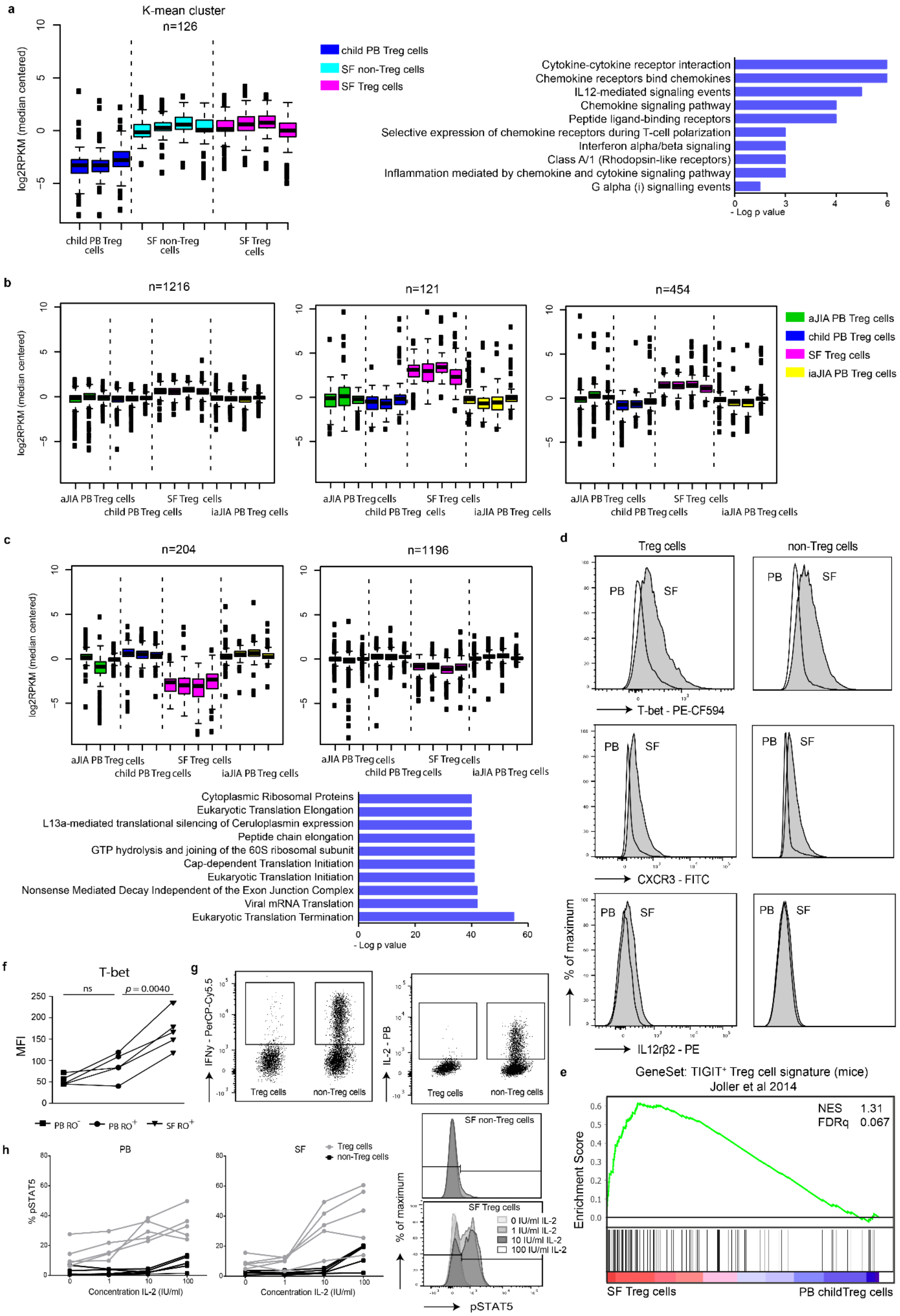
a
K-mean cluster
n=126
log2RPKM (median centered)
child PB Treg cells
SF non-Treg cells
SF Treg cells
Cytokine-cytokine receptor interaction
Chemokine receptors bind chemokines
IL12-mediated signaling events
Chemokine signaling pathway
Peptide ligand-binding receptors
Selective expression of chemokine receptors during T-cell polarization
Interferon alpha/beta signaling
Class A/1 (Rhodopsin-like receptors)
Inflammation mediated by chemokine and cytokine signaling pathway
G alpha (i) signalling events
- Log p value
b
n=1216
n=121
n=454
aJIA PB Treg cells
child PB Treg cells
SF Treg cells
iaJIA PB Treg cells
c
n=204
n=1196
Cytoplasmic Ribosomal Proteins
Eukaryotic Translation Elongation
L13a-mediated translational silencing of Ceruloplasmin expression
Peptide chain elongation
GTP hydrolysis and joining of the 60S ribosomal subunit
Cap-dependent Translation Initiation
Eukaryotic Translation Initiation
Nonsense Mediated Decay Independent of the Exon Junction Complex
Viral mRNA Translation
Eukaryotic Translation Termination
- Log p value
d
Treg cells
non-Treg cells
PB
SF
T-bet - PE-CF594
CXCR3 - FITC
IL12rβ2 - PE
% of maximum
e
GeneSet: TIGIT+ Treg cell signature (mice)
Joller et al 2014
NES 1.31
FDRq 0.067
Enrichment Score
SF Treg cells
PB childTreg cells
f
T-bet
ns
p = 0.0040
MFI
PB RO-
PB RO+
SF RO+
g
IFNγ - PerCP-Cy5.5
IL-2 - PB
Treg cells
non-Treg cells
h
PB
SF
% pSTAT5
Concentration IL-2 (IU/ml)
Treg cells
non-Treg cells
SF non-Treg cells
SF Treg cells
0 IU/ml IL-2
1 IU/ml IL-2
10 IU/ml IL-2
100 IU/ml IL-2
% of maximum
pSTAT5

Supplementary Figure 4. K-mean and GeneSet Enrichment analysis shows adaptation of SF Treg cells to Th1 environment. a K-mean analysis (k=14) on peripheral blood (PB) Treg cells, synovial fluid (SF) Treg cells and SF non-Treg cells (paired, n=4) with the cluster representing the genes upregulated in both SF Treg cells and non-Treg cells (n is the number of genes in the respective K-mean cluster; left) and the gene ontology pathway terms related to this cluster (right; top 10 ranked on p-value). Median+IQR with outliers are shown. **b** K-mean analysis (k=14) on all Treg cell groups derived from children (PB Treg cells from healthy children (n=3), juvenile idiopathic arthritis (JIA) patients with active (n=3) or inactive (n=4) disease and SF Treg cells from JIA patients (n=4)) with the K-mean clusters shown representing upregulated genes in SF Treg cells compared to the rest (left). Median+IQR with outliers are shown. **c** Similar as in **b** but concerning downregulated genes in SF Treg cells (top) and gene ontology pathway terms (bottom; top 10 ranked on p-value). **d** Representative MFI plots (for Fig. 3d) of T-bet (top), CXCR3 (middle) and IL12rβ2 (bottom) in Treg cells (left) and non-Treg cells (right), derived from paired SFMC (grey fill) and PBMC of JIA patients. The graphs are representative for two independent experiments with n=5 total. **e** GeneSet Enrichment Analysis (GSEA) of TIGIT$^+$ Treg cells signature genes (identified in mice by Joller *et al.*[20]) in pairwise comparisons involving SF and healthy child PB Treg cells, represented by the normalized enrichment score (NES) and FDR statistical value (FDRq, multiple hypothesis testing using sample permutation). **f** Comparison of the MFI of T-bet within PB CD45RO$^-$ (PB RO$^-$, square, healthy adults), PB CD45RO$^+$ (PB RO$^+$, circle, patients with active JIA), and SF CD45RO$^+$ (SF RO$^+$, triangle, patients with active JIA) (n=5 per subset). Statistical comparisons were performed using one-way ANOVA with Tukey correction for multiple testing. **g** Representative dotplots (for Fig. 3f) of IFNγ (left) and IL-2 (right) in SF Treg cells and non-Treg cells. The graphs are representative for two independent experiments. **h** Percentage and gating strategy (example of SF) of pSTAT5$^+$ cells in PB- and SF-derived Treg cells and non-Treg cells in presence of increasing IL-2 concentrations (n=5). Data are representative of two independent experiments. Source data are provided as a Source Data file and deposited under GSE161426.

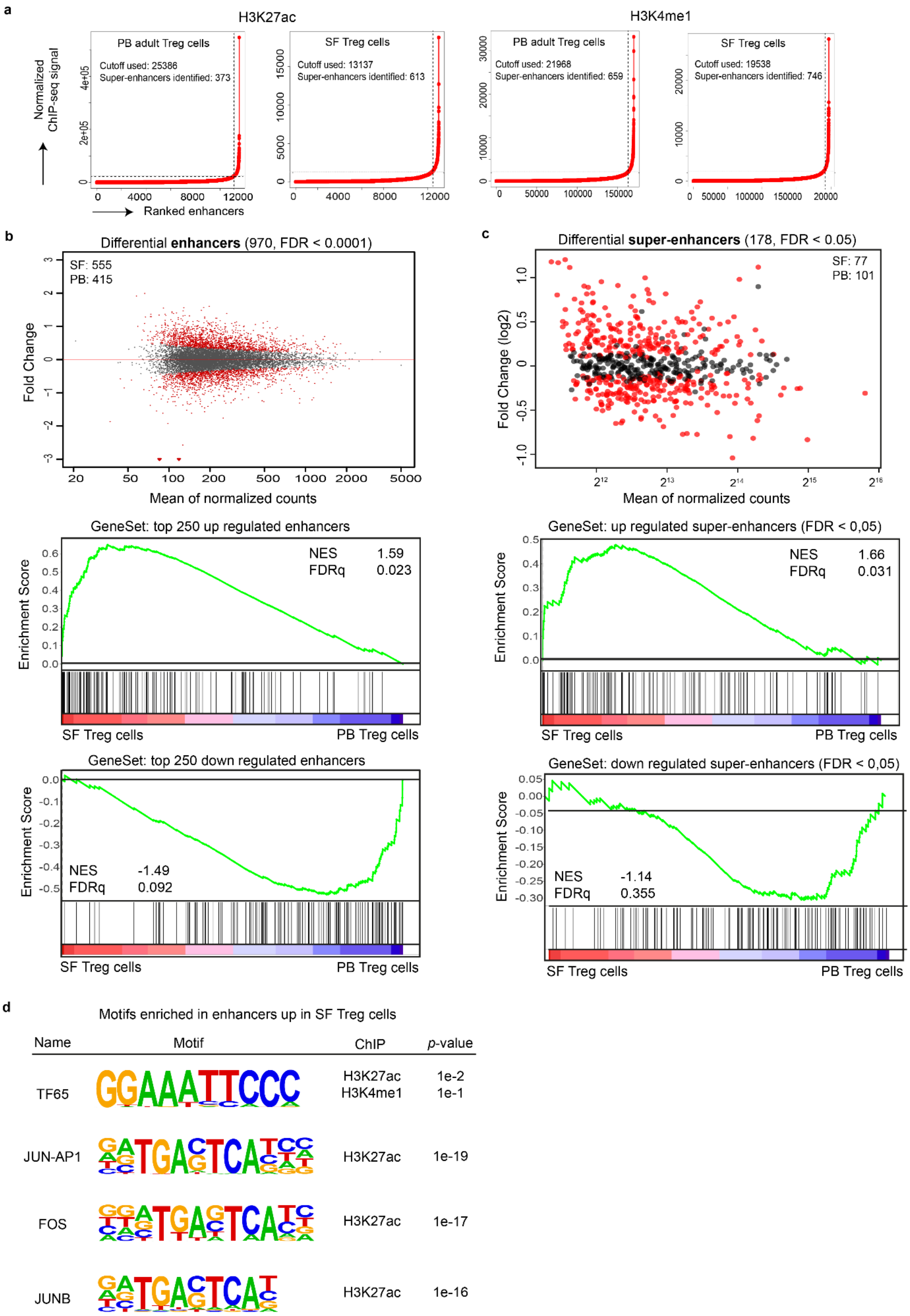

Supplementary Figure 5. Environment-specific effector Treg cells profile is regulated by the (super)enhancer landscape. a Representative examples of the normalized distribution of

H3K27ac (left two panels) and H3K4me1 (right two panels) ChIP-seq. The plots show the enhancers ordered on the respective histone marker signal performed with the ROSE algorithm; a line with a slope of one tangent to the curve is used as a cutoff to distinguish super-enhancers above and typical enhancers below the point of tangency. **b** MA plots of differentially expressed enhancers derived from H3K4me1 ChIP-seq (FDR <0.0001) in synovial fluid (SF) versus peripheral blood (PB) Treg cells with the number of SF- and PB-specific enhancers indicated (top). GeneSet Enrichment Analysis (GSEA) of the top 250 upregulated (middle) and downregulated (bottom) enhancers in pairwise comparisons involving transcriptome data of SF Treg cells and PB Treg cells derived from healthy adults, represented by the normalized enrichment score (NES) and the FDR statistical value (FDRq, multiple hypothesis testing using sample permutation). **c** Same as in **a** but for super-enhancers (FDR < 0.05). **d** Motifs, known and *de novo*, for transcription factor binding sites predicted using HOMER, enriched in the upregulated (super-)enhancers in SF Treg cells compared to healthy adult PB Treg cells for H3K27ac and H3K4me1 ChIP-seq. *p*-values: cumulative binomial distribution to calculate enrichment in target versus background sequences. Source data are deposited under GSE156426.

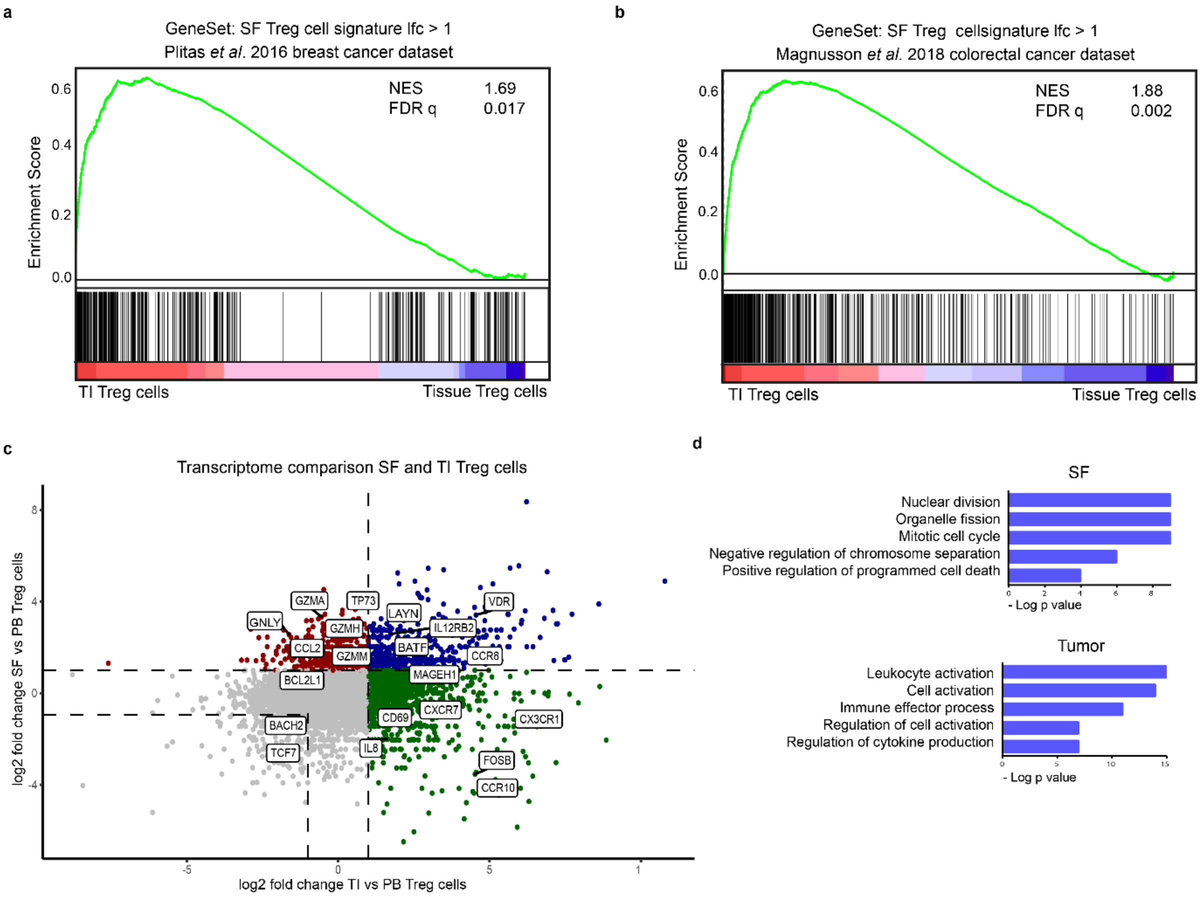

Supplementary Figure 6. The effector Treg cells program is universal and overlaps with the human tumor Treg cells signature. a GeneSet Enrichment Analysis (GSEA) of the differentially expressed genes (log2 fold change (lfc) >1, FDR <0.05) in synovial fluid (SF) compared to peripheral blood (PB) Treg cells in pairwise comparisons involving tumor-infiltrating (TI) Treg cells and normal tissue Treg cells derived from breast cancer tissue (Plitas *et al.*[16]), represented by the normalized enrichment score (NES) and FDR statistical value (FDRq, multiple hypothesis testing using sample permutation). **b** Same as in **a** but concerning colorectal cancer (Magnuson *et al.*[17]). **c** Comparison of transcriptomes between SF and PB Treg cells to TI and normal tissue Treg cells. The log2 fold change of SF to PB Treg cells (y-axis) versus TI to PB Treg cells (x-axis; derived from Plitas *et al.*[16]) is shown. Each dot represents a gene present in both datasets; in grey unchanged/downregulated in both effector Treg cells subsets compared to PB Treg cells, in blue upregulated in both SF and TI Treg cells, in red upregulated in SF Treg cells and in green upregulated in TI Treg cells; selected genes are highlighted. **d** Gene ontology biological process terms related to genes specifically upregulated in SF (left) or TI (right) Treg cells from Plitas *et al.*[16] compared to PB Treg cells in the respective datasets (healthy adults), ranked by enrichment scores. For all panels: SF Treg cells *n*=4, PB Treg cells *n*=4; Plitas TI Treg cells *n*=3, normal breast tissue Treg cells *n*=7; Magnuson TI Treg cells *n*=11 and normal colon tissue Treg cells *n*=7. Source data are deposited under GSE161426.

Supplementary Table 1. qPCR primer sequences.

Targets	
hGUSB_FW	CACCAGGGACCATCCAATACC
hGUSB_RV	GCAGTCCAGCGTAGTTGAAAAA
hCTLA4_FW	GGGGAATGAGTTGACCTTCCT
hCTLA4_RV	GGCACGGTTCTGGATCAATTA
hIL2RA_FW	CAATGCACAAGCTCTGCCACTC
hIL2RA_RV	ATCTGCCCCACCACGAAATGA
hTNFRSF8_FW	TTCTGGATGCAGGGCCAGT
hTNFRSF8_RV	CAGCTGCGTTGAGCTCCT
hFOXP3_FW	TCAAGCACTGCCAGGCG
hFOXP3_RV	CAGGAGCCCTTGTCGGAT
hIL10_FW	GAGGCTACGGCGCTGTCAT
hIL10_RV	CCACGGCCTTGCTCTTGTT
hIRF4_FW	AAGCCGGACCCTCCCACCTG
hIRF4_RV	TTGTGAACCTGCTGGGCTGGGA
hVDR_FW	TTCCGCTTCAGGATCATCTC
hVDR_RV	ACATCGGCATGATGAAGGA
hTBX21_FW	GGGAAACTAAAGCTCACAAAC
hTBX21_RV	CCCCAAGGAATTGACAGTTG
