# Supplementary material for: Conserved human effector Treg cell transcriptomic and epigenetic signature in arthritic joint inflammation
Source: Nat Commun. 2021 May 11;12:2710. doi: 10.1038/s41467-021-22975-7 (PMC8113485; doi:10.1038/s41467-021-22975-7)
Supplement: Supplementary file 3 — Description of Additional Supplementary Files [file 41467_2021_22975_MOESM3_ESM.pdf]

Description of Additional Supplementary Files

File Name: Supplementary Data 1

Description: Differentially expressed genes RNA-seq

File Name: Supplementary Data 2

Description: Differentially expressed enhancers and super-enhancers ChIP-seq

File Name: Supplementary Data 3

Description: Key-regulator analysis RNA-seq

File Name: Supplementary Data 4

Description: Gene set enrichment analysis Treg signatures

File Name: Supplementary Data 5

Description: Quality control data RNA- and ChIP-seq
