# Supplementary material for: Conserved human effector Treg cell transcriptomic and epigenetic signature in arthritic joint inflammation
Source: Nat Commun. 2021 May 11;12:2710. doi: 10.1038/s41467-021-22975-7 (PMC8113485; doi:10.1038/s41467-021-22975-7)
Supplement: Supplementary file 9 — Reporting Summary [file 41467_2021_22975_MOESM9_ESM.pdf]

nature research

Corresponding author(s): Femke van Wijk

Last updated by author(s): Feb 26, 2021

Reporting Summary

Nature Research wishes to improve the reproducibility of the work that we publish. This form provides structure for consistency and transparency in reporting. For further information on Nature Research policies, see our Editorial Policies and the Editorial Policy Checklist.

Statistics

For all statistical analyses, confirm that the following items are present in the figure legend, table legend, main text, or Methods section.

n/a	Confirmed	
☐	☒	The exact sample size (*n*) for each experimental group/condition, given as a discrete number and unit of measurement
☐	☒	A statement on whether measurements were taken from distinct samples or whether the same sample was measured repeatedly
☐	☒	The statistical test(s) used AND whether they are one- or two-sided<br>*Only common tests should be described solely by name; describe more complex techniques in the Methods section.*
☒	☐	A description of all covariates tested
☐	☒	A description of any assumptions or corrections, such as tests of normality and adjustment for multiple comparisons
☐	☒	A full description of the statistical parameters including central tendency (e.g. means) or other basic estimates (e.g. regression coefficient) AND variation (e.g. standard deviation) or associated estimates of uncertainty (e.g. confidence intervals)
☐	☒	For null hypothesis testing, the test statistic (e.g. *F*, *t*, *r*) with confidence intervals, effect sizes, degrees of freedom and *P* value noted<br>*Give P values as exact values whenever suitable.*
☒	☐	For Bayesian analysis, information on the choice of priors and Markov chain Monte Carlo settings
☒	☐	For hierarchical and complex designs, identification of the appropriate level for tests and full reporting of outcomes
☐	☒	Estimates of effect sizes (e.g. Cohen's *d*, Pearson's *r*), indicating how they were calculated

Our web collection on statistics for biologists contains articles on many of the points above.

Software and code

Policy information about availability of computer code

Data collection	FACS data were collected by FACS Canto II and Fortessa flow cytometer (BD Biosciences) with Diva Software v8.0 (BD Bioscience).<br>RNA-seq and ChIP-seq data were sequenced on a Nextseq500 platform (Illumina).<br>Microarray data were measured using a Human Gene 2.0 ST Array (Thermofisher).<br>qPCR data were measured using a QuantStudio 2 Real-Time PCR (ThermoFisher Scientific).
Data analysis	FACS data were analyzed on FlowJo Software v10 (Tree Star Inc.) and statistical analyses and visualisation were performed using Graphpad Prism 8.3.<br>RNA-seq was mapped using BWA (v0.7.5a, mem –t 7 –c 100 –M -R) and further analyzed using: R v3.2-v3.6 (DESeq2 v1.2 for differential gene expression, RegEnrich v1.0.0 for network analyses and custom scripts from https://github.com/mmokry/Mijnheer_Nat_Commun_2021 for K-means clustering), ToppFun for gene ontology analysis with default settings (https://toppgene.cchmc.org/enrichment.jsp), Morpheus software v0.1.1.1 for heatmap visualisation (https://software.broadinstitute.org/morpheus/) and GSEA software (v3.0 and v4.0.3, BroadInstitute) for gene set enrichment analysis.<br>ChIP-seq data was mapped with Bowtie 2.1.0 using default settings, samtools version 0.1.19 to convert SAM to BAM files, MACS-2.1.0 to identify enriched regions and further analyzed using: R v3.2-v3.6 (DiffBind v1.8.5 for differential binding analysis, ROSE algorithm to identify super-enhancers), BEDtools v2.17.0 for general manipulation of peak bed-files, HOMER software v4.11 for motif enrichment analysis, IGV-2.7.2 + igvtools-2.3.36 to visualize the tracks.<br>Microarray analysis: RMA algorithm for normalization and Qlucore Omics Explorer software v3.0 for gene expression analysis.<br>qPCR analysis: Fold change was calculated using the delta-delta Ct method (2^ΔΔCt) normalized to the housekeeping gene GUSB in Microsoft Excel 2016.

For manuscripts utilizing custom algorithms or software that are central to the research but not yet described in published literature, software must be made available to editors and reviewers. We strongly encourage code deposition in a community repository (e.g. GitHub). See the Nature Research guidelines for submitting code & software for further information.

Data

Policy information about availability of data

All manuscripts must include a data availability statement. This statement should provide the following information, where applicable:

- Accession codes, unique identifiers, or web links for publicly available datasets
- A list of figures that have associated raw data
- A description of any restrictions on data availability

The authors declare that all data supporting the findings of this study are available within the article, its Supplementary information files and the Source Data file. RNA-seq and ChIP-seq raw data is deposited under GSE161426 [https://www.ncbi.nlm.nih.gov/geo/query/acc.cgi?acc=GSE161426] and GSE156418 [https://www.ncbi.nlm.nih.gov/geo/query/acc.cgi?acc=GSE156418]. Publicly available ChIP-seq data for activated Treg cells, VDR and BATF was retrieved from GEO:GSE43119 [https://www.ncbi.nlm.nih.gov/gds/?term=GSE43119[Accession]], GSE89431 [https://www.ncbi.nlm.nih.gov/gds/?term=GSE89431[Accession]] and GSE32465 [https://www.ncbi.nlm.nih.gov/geo/query/acc.cgi?acc=GSM803538], respectively. A reporting summary for this Article is available as a Supplementary Information file.

Field-specific reporting

Please select the one below that is the best fit for your research. If you are not sure, read the appropriate sections before making your selection.

[x] Life sciences [ ] Behavioural & social sciences [ ] Ecological, evolutionary & environmental sciences

For a reference copy of the document with all sections, see nature.com/documents/nr-reporting-summary-flat.pdf

Life sciences study design

All studies must disclose on these points even when the disclosure is negative.

Sample size	Sample sizes were chosen based on prior research conducted in our laboratory (see also WIenke et al PMID: 32809975, Petrelli et al PMID: 30198907, Peeters et al PMID: 26387944) to provide sufficient patient/control samples in each group to provide informative results and perform statistical testing, accounting for variability in possible cell isolation, sample storage or patient characteristics.
Data exclusions	No complete samples were excluded, flow subgated samples were not taken into account when the population counted < 100 cells in total.
Replication	We performed flow analysis in at least two independent experiments and observed similar results.
Randomization	No randomization was performed for flow cytometry nor ChIP-seq and RNA-seq. The aim was to investigate the populations of interest disregardful of the patient/control characteristics. Patients and controls only fall into one of the subcategories measured, so here randomization was not applicable. For selection of the participants to be included for measurment the largest and most recently stored samples were included to ensure optimal quality of the samples.
Blinding	There was no blinding for the identity of the samples. Compared samples were collected and analyzed under the same conditions. To avoid introducing bias due to not blinding, samples were measured in a standardized way, in batches consisting of samples from all comparison groups.

Reporting for specific materials, systems and methods

We require information from authors about some types of materials, experimental systems and methods used in many studies. Here, indicate whether each material, system or method listed is relevant to your study. If you are not sure if a list item applies to your research, read the appropriate section before selecting a response.

Materials & experimental systems

n/a	Involved in the study	
[ ]	[x]	Antibodies
[x]	[ ]	Eukaryotic cell lines
[x]	[ ]	Palaeontology and archaeology
[x]	[ ]	Animals and other organisms
[ ]	[x]	Human research participants
[x]	[ ]	Clinical data
[x]	[ ]	Dual use research of concern

Methods

n/a	Involved in the study	
[ ]	[x]	ChIP-seq
[ ]	[x]	Flow cytometry
[x]	[ ]	MRI-based neuroimaging

Antibodies

Antibodies used	Viability Dye eFluor 506 (1:1000, eBioscience, cat # 65-0866-14, lot # 1923275)

anti-human CD3 AF700 (clone UCHT1, 1:50, Biolegend, cat # 300424, lot # B279942)
anti-human CD4 BV785 (clone OKT4, 1:100, Biolegend, cat # 317442, lot # B282604)
anti-human CD127 BV605 (clone A019D5, 1:50, Sony Biotechnology, cat # 2356670, lot # 156361)
anti-human CD25 BV711 (clone 2A3, 1:50, BD, cat # 563159, lot # 9081985)
anti-human FOXP3 eF450 (clone PCH101, 1:50, eBioscience, cat # 48-4776-42, lot # 1946678)
anti-human FOXP3 APC (clone PCH101, 1:25, eBioscience, cat # 17-4776-42, lot # 4293548)
anti-human VDR PE (clone D-6, 1:100, Santa Cruz Biotechnology, cat # sc-13133, lot # K2917)
anti-human CD3 BV510 (clone OKT3, 1:400, Biolegend, cat # 317332, lot # B210180)
anti-human CD25 PE-Cy7 (clone MA251, 1:25, BD, cat # 557741, lot # 5135905 & 4189778)
anti-human CD4 FITC (clone RPA-T4, 1:200, eBioscience, cat # 11-0049-42, lot # E10610-1637)
anti-human CD127 AF647 (clone HCD127, 1:50, Biolegend, cat # 2356590, lot # 92612 & 120730)
anti-human CD3 PerCP-Cy5.5 (clone UCHT1, 1:100, Biolegend, cat # 300430, lot # B193956)
anti-human CD8 APC (clone SK1, 1:100, BD, cat # 345775, lot # 6013777)
anti-human T-bet eF660 (clone eBio4B10, 1:50, eBioscience, cat # 50-5825-82, lot # E12135-1630)
anti-human pSTAT5-PE (clone pY695, 1:25, BD, cat # 612567, lot # 3319802 & 4296746)
anti-human CD4 PerCP-Cy5.5 (clone SK3, 1:200, BioLegend, cat # 344608)
anti-human CD45RA APC-Cy7 (clone HI100, 1:50, BioLegend, cat # 304128)
anti-human CD45RO PB (clone UCHL1, 1:50, BioLegend, cat # 304218)
anti-human CD127 FITC (clone A019D5, 1:50, BioLegend, cat # 351312)
anti-human CD25 PE (clone REA945, 1:50, Miltenyi Biotec, cat # 130-115-534)
anti-human CD4 APC-eFluor780 (clone RPA-T4, 1:50, eBioscience, cat # 47-0049-42)
anti-human CD127 BV421 (clone HIL-7R-M21, 1:40, BD, cat # 562436)
anti-human CTLA-4 PE (clone BNI3, 1:12.5, BD, cat # 555853)
anti-human TIGIT PerCP-eFluor710 (clone MBSA43, 1:50, eBioscience, cat # 46-9500-42)
anti-human PD-1 BV711 (clone EH12.1, 1:100, BD, cat # 564017)
anti-human GITR FITC (clone #110416, 1:8.3, R&D, cat # FAB689F)
anti-human IL12RB2 (clone 2B6/12β2, 1:5, BD, cat # 550723)
anti-human T-bet PE-CF594 (clone 04-46, 1:25, BD, cat # 562467)
anti-human IFNγ PerCP-Cy5.5 (clone 4S.B3, 1:40, BD, cat # 45-7319-42)
anti-human CD127 PerCP-Cy5.5 (clone HCD127, 1:25, Biolegend, cat # 317610)
anti-human CD127 PE-Cy7 (clone HCD127, 1:50, Biolegend, cat # 25-1278-42)
anti-human FOXP3 PE-CF594 (clone 259D/C7, 1:50, BD, cat # 562421)
anti-human ICOS APC (clone ISA3, 1:20, eBioscience, cat # 17-9948-42)
anti-human CXCR3 FITC (clone G025H7, 1:40, Biolegend, cat # 353704)
anti-human IL2 PB (clone MQ1-17H12, 1:100, Biolegend, cat # 500324)
anti-human CTLA-4 APC (clone BNI3, 1:12.5, BD, cat # 555855)
anti-human CD4 APC-Cy7 (clone RPA-T4, 1:100, Biologend, cat # 300518)
anti-human CD30 FITC (clone Ber-H8, 1:12.5, BD, cat # 555829)
anti-human FOXP3 PerCp-Cy5.5 (clone PCH101, 1:50, eBioscience, cat# 45-4776-42)

Validation

All antibodies used are standard antibodies and have been validated on human peripheral blood lymphocytes by the manufacturers.
There were no validation statements regarding the antibodies on the websites of the manufacturers.
The antibodies were titrated in house on human peripheral blood lymphocytes.

Human research participants

Policy information about studies involving human research participants

Population characteristics	Pediatric patients with oligo-articular juvenile idiopathic arthritis and adult patients with rheumatoid arthritis with no use of corticosteroids in the 3 months prior to sample collection. Other patient characteristics were not taken into account. If available paired peripheral blood and synovial fluid samples were taken. Healthy adult and child peripheral blood was collected without any prior selection on characteristics.<br>JIA patients and healthy children were age-matched.<br>Of the JIA patients n=8 were diagnosed with extended oligo JIA and n=33 with oligo JIA, according to the revised criteria for JIA83, with an average age of 11.3 years (range 3.2-19 years) and a disease duration at the time of inclusion of 4.9 years (range 0.1-15 years). The average age of RA patients (n = 7) was 61 years (range 30-75 years). For healthy controls (n=20) the average age was 41.7 years with range 27-62 years. For healthy children (n = 8) the average age was 11.4 years with range 7.3-15.6 years (age-matched to the JIA patients).
Recruitment	All patients presenting in the hospital receiving a diagnosis of (extended) oligo-articular juvenile idiopathic arthritis or rheumatoid arthritis were asked to participate in the respective biobanking protocols. Healthy child control samples were included from children undergoing elective surgery and healthy adult controls are employees of the University Medical Center Utrecht. All material was stored in the biobank until use and samples were selected with help of the treating physician based on: for pediatric patients having oligo-articular juvenile idiopathic arthritis and adults with rheumatoid arthritis with no use of corticosteroids in the 3 months prior to sample collection, for healthy controls PBMC from adults and children were randomly selected from the available database based on availability of enough stored cells. Healthy child controls were age-matched with the juvenile idiopathic arthritis patients, and if available paired peripheral blood and synovial fluid was requested from both the juvenile idiopathic arthritis and rheumatoid arthritis patients. There was no selection based on other patient characteristics. There is no (self-)selection bias since participants were taken randomly from the biobank. The measurements were standardized and objective and therefore not introducing confirmation bias. Other types of research bias did not influence this study.
Ethics oversight	All procedures were in accordance to institutional guidelines and were approved by the Institutional Review Board of the University Medical Center Utrecht (approval no. 11-499/C; JIA, approval no. 05-149/K; healthy children and Mini Donor Service) and the Bromley Research Ethics Committee (approval no. 06/Q0705/20; RA). Informed consent was obtained from all the participants and/or from their parents/guardians/legally authorized representatives.

Note that full information on the approval of the study protocol must also be provided in the manuscript.

ChIP-seq

Data deposition

- [x] Confirm that both raw and final processed data have been deposited in a public database such as GEO.
- [x] Confirm that you have deposited or provided access to graph files (e.g. BED files) for the called peaks.

Data access links<br>*May remain private before publication.*	GEO accession GSE156418
Files in database submission	PBTregH1_H3JM5BGXX-_S2.merged.bam.sorted.bam<br>PBTregH2_H3JM5BGXX-_S2.merged.bam.sorted.bam<br>PBTregH3_H3JM5BGXX-_S2.merged.bam.sorted.bam<br>SFTregH1_H3JM5BGXX-_S2.merged.bam.sorted.bam<br>SFTregH2_H3JM5BGXX-_S2.merged.bam.sorted.bam<br>SFTregH3_H3JM5BGXX-_S2.merged.bam.sorted.bam<br>PBTregH1_H3JM5BGXX-_S2.merged.bam_peaks.narrowPeak<br>PBTregH2_H3JM5BGXX-_S2.merged.bam_peaks.narrowPeak<br>PBTregH3_H3JM5BGXX-_S2.merged.bam_peaks.narrowPeak<br>SFTregH1_H3JM5BGXX-_S2.merged.bam_peaks.narrowPeak<br>SFTregH2_H3JM5BGXX-_S2.merged.bam_peaks.narrowPeak<br>SFTregH3_H3JM5BGXX-_S2.merged.bam_peaks.narrowPeak<br>PBTregH1_H3JM5BGXX-_S2.merged.bam_summits.bed<br>PBTregH2_H3JM5BGXX-_S2.merged.bam_summits.bed<br>PBTregH3_H3JM5BGXX-_S2.merged.bam_summits.bed<br>SFTregH1_H3JM5BGXX-_S2.merged.bam_summits.bed<br>SFTregH2_H3JM5BGXX-_S2.merged.bam_summits.bed<br>SFTregH3_H3JM5BGXX-_S2.merged.bam_summits.bed<br>PBTregD1_dedup.bam<br>PBTregD2_dedup.bam<br>PBTregD3_dedup.bam<br>SFTregD1_dedup.bam<br>SFTregD2_dedup.bam<br>SFTregD3_dedup.bam<br>PBTregD1_dedup.unique_peak.cod

PBTregD2_dedup.unique_peak.cod
PBTregD3_dedup.unique_peak.cod
SFTregD1_dedup.unique_peak.cod
SFTregD2_dedup.unique_peak.cod
SFTregD3_dedup.unique_peak.cod

Genome browser session (e.g. UCSC)

Upon request we can share the TDF files for visualization in IgV.

Methodology

Replicates

Both H3K4me1 and H3K27ac ChIP-seq 3 biological replicates for both healthy controls and juvenile idiopathic arthritis patients.

Sequencing depth

Sample ID Raw reads (#) Mapped reads (%) Peaks called (#) Length
PBTreg D1 68837821 84.0 5526 single-end 75bp
PBTregD2 59317309 86.2 7665 single-end 75bp
PBTregD3 41033751 86.3 12537 single-end 75bp
SFTregD1 50555067 87.6 17170 single-end 75bp
SFTregD2 54678376 85.9 13810 single-end 75bp
SFTregD3 53170500 88.2 9963 single-end 75bp
PBTreg H1 17314880 75.0 55519 single-end 75bp
PBTregH2 9982870 75.0 50941 single-end 75bp
PBTregH3 23282682 73.1 76573 single-end 75bp
SFTregH1 11703191 76.1 42695 single-end 75bp
SFTregH2 16982284 76.5 47079 single-end 75bp
SFTregH3 17974573 76.8 48027 single-end 75bp

Antibodies

anti-histone H3 acetyl K27 antibody (ab4729; Abcam)
anti-histone H3 (mono methyl K4) antibody (ab8895; Abcam)

Peak calling parameters

Reads were mapped to the reference genome hg19 with Bowtie 2.1.0 for H3K27ac ChIP-seq and to hg38 with BWA for H3K4me1 ChIP-seq using default settings .
Peaks were called using MACS-2.1.0. Enriched regions were identified compared to the input control using MACS2 callpeak--nomodel --exttsize 300 --gsize=hs -p 1e-9.

Data quality

To ensure that our data were of high quality and reproducibility, we called peaks with enrichment > 5-fold over control and compared the peak sets using the ENCODE overlap rules. We also used the FASTQC pipeline to measure the data quality.

Software

Mapping: BWA for H3K4me1 and Bowtie 2.1.0 for H3K27ac
Peak-calling: MACS-2.1.0

Flow Cytometry

Plots

Confirm that:

- [x] The axis labels state the marker and fluorochrome used (e.g. CD4-FITC).
- [x] The axis scales are clearly visible. Include numbers along axes only for bottom left plot of group (a 'group' is an analysis of identical markers).
- [x] All plots are contour plots with outliers or pseudocolor plots.
- [x] A numerical value for number of cells or percentage (with statistics) is provided.

Methodology

Sample preparation

SF of JIA and RA patients was incubated with hyaluronidase (Sigma-Aldrich) for 30 min at 37°C to break down hyaluronic acid. Synovial fluid mononuclear cells (SFMCs) and peripheral blood mononuclear cells (PBMCs) were isolated using Ficoll Isopaque density gradient centrifugation (GE Healthcare Bio-Sciences, AB) and frozen in Fetal Calf Serum (FCS) (Invitrogen) containing 10% DMSO (Sigma-Aldrich) until use. Cells were thawed, plated in a 96-wells plate and stained with the surface antibodies for 25 min at 4C. For intracellular staining the Intracellular Fixation & Permeabilization Buffer Set (eBioscience) was used according to the manufacturer's protocol, followed by incubation with intracellular antibodies at 4C. For intracellular cytokine production, cells were first stimulated for a total of 4 hours with PMA (20 ng/ml; MP Biomedicals) and ionomycin (1 µg/ml; Calbiochem). Golgi stop (1/1500; BD Biosciences) was added for the last 3 hours of stimulation.
For the suppression assay: Total PBMC were labeled with 2µM ctViolet (Thermo Fisher) and cultured alone or with different ratios of sorted Treg (1:16, 1:8, 1:4, 1:2). Cells were cultured in RPMI1640 media containing 10% human AB serum with addition of L-Glutamine and Penicillin/Streptomycin. PBMC were stimulated by 0,1 µg/ml coated anti-CD3 (eBioscience) and incubated for four days in a 96 well round bottom plate (Nunc) at 37˚C. After 4 days cells were stained as described above.
FOr the vitamin D incubation assay: orted Tregs (CD3+CD4+CD25+CD127low, 50.000) were plated in a round bottom 96-wells

	plate in the presence of 10% human AB serum, anti-CD3/CD28 (Dynabeads Human T-activator CD3/CD28, ThermoFisher Scientific) at a 1:5 ratio (1 bead to 5 cell ratio), 100 IU/ml (h)IL-2 (Proleukin; Novartis) for 2 days. In addition, 0 (control), 1, 10 or 100 nM 1α,25-Dihydroxyvitamin D3 (Sigma-Aldrich) was added. Staining as described above.
Instrument	LSRFortessa or FACSCanto II cytometers were used for cell acquisition and a FACS Aria III was used for cell sorting (all BD Bioscience)
Software	Data were acquired with Diva Software v8.0 (BD Bioscience) and analyzed with FlowJo software v10 (TreeStar).
Cell population abundance	All sorted populations reached a purity > 99%.
Gating strategy	Gates were set based on FMO's if required. The gating strategies are depicted in the supplementary figures.

☒ Tick this box to confirm that a figure exemplifying the gating strategy is provided in the Supplementary Information.
